# Positive Subjective Health of Older Homeless and Older Irish Travelers: a Participant-Led Approach

**DOI:** 10.1093/geroni/igaa057.353

**Published:** 2020-12-16

**Authors:** Bridin Carroll, Kieran Walsh

**Affiliations:** National University of Ireland Galway, Galway, Galway, Ireland

## Abstract

Older people experiencing homelessness and older Irish Travellers (OTOH) are both over-represented in the cohort who use acute health services. Impending health care reform in Ireland will be based on primary care models, meaning home and community care will be, for the first time, underpinned by a regulatory framework. For these reasons, this study aims to gain a nuanced understanding of how OTOH, as marginalised older people, might be best served by new home care and community care models. Using a qualitative, voice-led approach, a life course and structural determinants lens is employed to probe the health conditions, experiences and expectations of OTOH, as well as their perceptions and values around the concept of ‘home’. The research processes and outcomes of one of five phases of research are presented in this paper: participant-led research. In this phase, five OTOH were trained and assisted to complete a short research project which fed into the goals of the wider study. Emergent findings suggest that social connections underpin health and well-being for OTOH, throughout the life course, and presently. This was also seen as a fundamental element for healthy and positive ageing. In addition, ‘home’ was defined with reference to the presence (or absence) of familial or other social connections. This study represents an important contribution to scholarship on old age social exclusion. It is entirely novel in its approach to focusing on OTOH health and wellbeing. The outputs of this study also have important implications for upcoming health reform policies in Ireland.

